# Feasibility and effect of cognitive-based board game and multi-component exercise interventions on older adults with dementia

**DOI:** 10.1097/MD.0000000000038640

**Published:** 2024-06-28

**Authors:** Hui-Wen Chang, Guey-Hau Wu

**Affiliations:** a Department of Nursing, Taipei Veterans General Hospital, Taipei, Taiwan; b National Taipei University of Nursing and Health Sciences, School of Nursing, Taipei, Taiwan.

**Keywords:** board game and multi-component exercise, cognitive-based board game, dementia, dementia fall risk

## Abstract

**Background:**

Taiwan is an aging society, and the number of people with dementia is rapidly increasing. Due to a decline in cognitive and physical function, older adults with dementia not only gradually lose the ability to complete daily living tasks on their own, but are also at a higher risk of falls and injurious falls. It is important to develop interventions that combine cognitive and exercise training for older adults with dementia to promote or maintain their cognitive and physical functions and reduce their risk of falls. This study aimed to investigate the feasibility and effect of cognitive-based board games and multi-component exercise interventions on cognitive function, physical fitness, and fall risk in older adults with dementia.

**Methods:**

This was a quasi-experimental study with a single-group pretest and post-test design. The study participants were 41 community-dwelling older adults with mild to moderate dementia. They received cognitive-based board games and multi-component exercise interventions once a week for 12 weeks. The interventions included 1 hour of exercise training and 1 hour of cognitive training. Scores for the Taiwan version of the Montreal Cognitive Assessment (MoCA-T), physical fitness, and the St. Thomas Risk Assessment Tool for Falling Elderly Inpatients (STRATIFY) were measured as outcome indicators at baseline and after the 12-week period.

**Results:**

The overall MoCA-T score increased significantly (effect size = 0.402), with participants with mild dementia showing a greater increase (effect size = 0.522) than those with moderate dementia (effect size = 0.310). Participants’ physical fitness performance improved. Female participants exhibited significant improvements in the 30-second chair stand test (effect size = 0.483) and 8-foot up-and-go test (effect size = 0.437). The fall risk score decreased by 0.05 points, the change was not significant.

**Conclusion:**

The cognitive-based board game and multi-component exercise interventions used in this study are beneficial for improving cognitive function and physical fitness in older adults with dementia. These interventions are feasible and suitable for promotion among community-dwelling and institution-dwelling older adults with mild cognitive impairment or dementia to delay the decline in cognitive and physical function.

## 1. Introduction

The number of people with dementia in Taiwan is increasing annually. By the end of December 2022, it was estimated that there were more than 307,931 people with dementia, accounting for 7.54% of Taiwan’s elderly population aged 65 years and over,^[1]^ a percentage higher than the worldwide prevalence rate of dementia (6.9%) among adults aged 65 years and over estimated by the WHO in 2019, and among those in Africa (4.44%), Southeast Asia (4.04%), and the Eastern Mediterranean Region (5.89%).^[2]^ These results indicate that medical treatment and care of older adults with dementia are important global issues.

Studies of various non-drug interventions for dementia have gradually gained attention in various countries. Cognitive intervention enables older adults with dementia to continuously practice and use various cognitive abilities in activities, including attention, memory, language, executive function, and logical reasoning, which primarily aims to help older adults with dementia maintain their current cognitive function and delay degeneration.^[3,4]^ Exercise interventions are beneficial for brain and cognitive function. Exercise not only improves risk factors for dementia, such as diabetes mellitus, hypertension, obesity, and depression, but also plays a role in protecting neurogenesis in the brain and promoting neuroplasticity.^[5]^ Fall prevention in older adults is an important public health issue worldwide. The risk of falls due to poor balance or muscle strength in older adults with dementia is twice that in healthy older adults.^[6]^ Multi-component exercise interventions can improve the physical fitness of older adults with dementia, maintain their independent life skills, increase their balance and movement abilities, slow the decline in their physical activity, and reduce the risk of needing medical care due to falls.^[7]^

The course of dementia has lasted several decades. When older adults with dementia begin to experience declines in cognitive and physical function, their functional needs for mobility and fall risk reduction increase, and caregivers face more challenges.^[8,9]^ In 2017, the Ministry of Health and Welfare in Taiwan set up community-based “Integrated Dementia Care Centers” and “Community-based Dementia Service Stations” in each county and city; community-based dementia service stations provide care, caregiver support and other diversified services for older adults with dementia, including cognitive function activities, social interaction, shared meals, and care resources and consultation services for caregivers, to reduce the burden of care.^[10]^

Board games are diverse and easy to operate, making them suitable for all age groups. Therefore, community-based dementia service stations in Taiwan often use board games as a group activity for cognitive intervention. Compared to other types of games, board games provide participants with specific experiences, cognitive stimulation, instant feedback, and social interaction. The diversity of board games encourages older adults to participate in community courses. Therefore, board games are often used to prevent cognitive decline in Taiwan.^[11]^

In terms of delaying deterioration from dementia, the implementation of cognitive or exercise intervention alone is not as effective as activities that combine cognitive and exercise interventions.^[12]^ The combination of cognitive and exercise interventions is conducive to maintaining and improving cognitive function and physical fitness in older adults with dementia.^[13]^ Therefore, this study aimed to investigate the feasibility and effect of cognitive-based board games and multi-component exercise interventions on cognitive function, physical fitness, and fall risk in older adults with dementia.

## 2. Methods

### 2.1. Study design

This study adopted a quasi-experimental design, and conducted qualitative interviews. The population of older adults with dementia is relatively small and recruitment is challenging. Therefore, a single-group pretest and post-test method was employed. According to Karssemeijer et al,^[12]^ exercise and cognitive dual-task interventions can delay the decline in cognitive function in older adults with dementia. A moderate effect size was used in the present study. Therefore, G*Power software (V.3.1.9.4) was used to calculate the sample size needed for the study using a two-tailed test, with α set to 0.05, the power set to 0.8, and the effect size set to moderate (Cohen’s *d* = 0.5),^[14]^ the required sample size was calculated to be 34 participants. Considering a loss rate of 20%, the goal was to recruit at least 41 participants for the study.

### 2.2. Participants and recruitment

This study was conducted at 5 community-based dementia service stations in northern Taiwan, between 2021 and 2022. Community-based dementia service stations are designed to serve individuals aged 65 years and above who have been diagnosed with dementia. Participants were mainly referred from hospitals and recruited from the community based on the eligibility criteria. Therefore, this study included people aged 65 years and above, diagnosed with mild (CDR = 1) or moderate dementia (CDR = 2), able to participate in exercise activities, and able to communicate in Mandarin and express their intentions clearly. The exclusion criteria were participants who had contraindications for exercise due to disease factors. Fifty participants met the inclusion criteria, completed the written consent form, and completed the pretest. However, during the study period, 9 participants were lost: 7 dropped out of the study due to illness, and 2 were absent too many times and failed to complete the post-test. Therefore, in this study, 41 participants completed the 12-week intervention as well as the pretest and post-test.

### 2.3. Cognitive-based board game and multi-component exercise interventions

In this study, a multi-component group interaction intervention that integrated cognitive-based board games and multiple types of exercises was developed for older adults with dementia. Interventions were conducted once a week for 12 weeks. Each session included a 1-hour exercise intervention and a 1-hour cognitive intervention. The participants underwent interventions in a group setting, with a maximum of 15 participants in each group. A licensed occupational therapist led the multi-component exercise intervention program. The cognitive-based board game intervention was led by a professional trainer from a board game company with extensive experience facilitating games for community-dwelling older adults. A cognitive-based board game and multi-component exercise intervention schedule was developed based on a literature review and referencing research intervention measures in various countries (Table 1).

**Table 1:** Curriculum schedule for the cognitive and exercise interventions.

Week	Multi-component exercise intervention	Cognitive intervention	
	Multi-component exercise included warm-up, aerobic exercise (4 sets of 8 reps for each movement), muscle strength training, balance training, and relaxation	Board game name	Dimensions of cognitive training
1	14 aerobic exercise movements; muscle strength training with a gym ball: 8 upper-limb movements, 2 core-muscle movements, and 4 lower-limb movements; and chair exercises for balance training.	Dementia Out	Visuospatial executive ability (color pairing), memory, and attention.
2		Let’s Bandoh	Visuospatial executive ability (visual seeking), naming, and abstraction
3	14 aerobic exercise movement; muscle strength training with a gym ball: 8 upper-limb movements, 4 core-muscle movements, and 5 lower-limb movements; and chair exercises for balance training.	Feed Us	Visuospatial executive ability (color), memory (auditory memory), attention, and abstraction.
4		Five Little Fish	Visuospatial executive ability (color pairing), memory, and attention
5	16 aerobic exercise movement; muscle strength training with a resistance band: 5 upper-limb movements, 4 core-muscle movements, and 2 lower-limb movements; and chair exercises and standing exercises for balance training.	Noah’s Ark	Executive ability, naming, attention, and abstraction (logic).
6		Toddles-Bobbles	Visuospatial executive ability, naming, memory, language ability, and abstraction.
7	18 aerobic exercise movements; muscle strength training with a resistance band: 5 upper-limb movements, 4 core-muscle movements, and 2 lower-limb movements; and standing exercises for balance training.	Chromino	Visuospatial executive ability (color pairing), attention, and abstraction.
8		Matryoshkaville	Visuospatial executive ability (color), naming, memory, and language ability.
9	20 aerobic exercise movements; muscle strength training with a resistance band: 7 upper-limb movements, 4 core-muscle movements, and 4 lower-limb movements; and standing exercises for balance training with a gym ball.	Stick Stack	Executive ability, attention, and abstraction.
10		Legend of the wishing table	Executive ability, attention, and language ability.
11	20 aerobic exercise movements; muscle strength training with a resistance band: 7 upper-limb movements, 4 core-muscle movements, and 4 lower-limb movements; and standing exercises for balance training with a gym ball.	BLINK	Visuospatial executive ability (color), attention, and abstraction.
12		Rummikub	Visuospatial executive ability (color), attention, abstraction.

In this study, a multi-component exercise intervention was designed to enhance health and physical fitness^[15]^ with the intensity gradually increasing every week. The following movements were performed: 1. warm-up; 2. main exercises: aerobic exercise (stepping in a sitting position and changing foot movements in coordination with hand movements), muscle strength training (using a resistance band or gym ball to train the muscles of the upper and lower limbs and the core muscles), and balance coordination training (balance training movements in the sitting or standing position); and 3. relaxation and stretching. For muscle strength training, a gym ball was used for the 1st to 4th weeks, and a resistance band was used for the 5th to 12th weeks. For balance coordination training, a gym ball was used for the 9th to 12th weeks. The resistance bands and gym balls were of the same brand and model, respectively. For balance coordination training, an armchair was used in the first week, the sit-to-stand method was gradually introduced in the 7th week, and standing balance training was introduced in the 9th week.

In many countries, technology-based interventions are often used in cognitive training, and individual interventions are common.^[12,16,17]^ However, non-technical group interactions are mostly used for cognitive interventions in Taiwan.^[11,18]^ Considering that the study sites were dementia service stations, which were suitable for group interaction interventions, cognitive interventions were mainly conducted through non-technological group board games. The cognitive intervention in this study was designed based on the 7 dimensions of cognition tested in the Taiwan version of the Montreal Cognitive Assessment (MoCA-T) and interesting board games. Through a different board game every week, cognitive interventions focused on visuospatial executive ability, naming, memory, attention, language ability, abstraction, and orientation (Table 1). In addition, in each class, a calendar with large characters was used to introduce the year, month, and day to the participants to provide a sense of orientation.

To determine the suitability of the cognitive-based board game and multi-component exercise intervention design, 3 board game experts and 2 exercise experts for older adults were invited to examine and evaluate the content validity based on whether the weekly curriculum design met the criteria for older adults with dementia and was suitable for their cognitive and physical fitness levels. The content validity index values for the cognitive-based board game intervention content and the multi-component exercise intervention content were 0.89 and 0.92, respectively, with both exceeding the requirement of 0.8 or above. At each study site, one trainer served as a cognitive intervention, and one trainer served as an exercise intervention for a total of 12 weeks. To ensure consistency of the intervention content and methods, trainers attended a training session before the intervention.

The researchers compiled manuals outlining the content to be covered in each session, adhering to the recommendations of 5 experts in a cognitive-based board game and multi-component exercise intervention design. The cognitive-based board game manual includes explanations of the game’s components and rules, starting with simple instructions and progressing to advanced gameplay strategies. The final section consisted of the participants’ reflections and sharing of experiences throughout the 12 sessions. The exercise manual included counts, repetitions, and sequence of each exercise movement over the 12 sessions. Furthermore, posters illustrating exercise movements were displayed for both trainers and participants during each session. One researcher supervised the trainers to ensure adherence to the manual, thus ensuring consistency and fidelity of the interventions.

### 2.4. Data collection

The staff collected baseline data 1 week before the start of the interventions (T0) and posttest data 1 week after the end of the interventions in week 12 (T1). The staff included 3 nurses with 4 to 12 years of work experience. They completed examiner consistency training 1 week before data collection. The collected data included basic demographic data, health status and behavior, MoCA-T score, 3 physical fitness items, and the St. Thomas Risk Assessment Tool in Falling Elderly Inpatients (STRATIFY) score. The test duration was approximately 15 to 20 minutes. Additionally, the researcher recorded the attendance and participation of the enrolled participants during each session and collected qualitative data through interactions with the participants and primary caregivers. The staff members were blinded to the intervention.

### 2.5. Instrument

The MoCA-T was used to measure cognitive function. Three physical fitness movements were used to measure the upper- and lower-limb muscle strength and balance status of the participants. Fall risk was measured using the STRATIFY tool.

#### 2.5.1. The Taiwan version of Montreal Cognitive Assessment (MoCA-T)

MoCA was developed by Nasreddine et al^[19]^ and has been used in more than 200 countries worldwide. It is more sensitive than the Mini-Mental State Examination for detecting executive and cognitive function. Multilingual versions of the MoCA are available for downloading at www.mocacognition.com, which is a short and rapid tool for screening for cognitive abnormalities and measuring 7 cognitive dimensions: visuospatial execution, naming, memory (immediate and delayed memory), attention, language ability, abstraction, and orientation. The maximum score on the scale is 30 points. If a participant’s educational attainment was below the high school level, one point was added to the total score. Scores < 26 points indicated cognitive impairment and scores ≥ 26 points indicated normal cognitive function. The sensitivity and specificity of the MoCA-T for identifying mild cognitive impairment were 92% and 78%, respectively.^[20,21]^

#### 2.5.2. Hand grip

Hand grip strength was evaluated using standard TTM hand grip dynamometers (TTM-YO) to measure the muscle strength of the upper limbs of the participants. The measurement data for the left and right hands were averaged. Grip strength was measured in kilograms, with a larger numerical value indicating better upper limb muscle strength.

#### 2.5.3. 30-second chair stand test

Lower limb muscle strength was measured using the 30-second chair stand test. Each participant sat in the center of a stable chair with their feet on the floor and hands crossed in front of their chest. The number of times the participant got up and sat down within 30 seconds was counted; a larger numerical value indicated better lower limb muscle strength.

#### 2.5.4. 8-foot up-and-go test

Agility and dynamic balance were measured using the 8-foot up-and-go test. The test measures the number of seconds required for a participant to get up from a chair, walk around an obstacle cone 8 feet in front of him or her, return to the chair, and sit down. The shorter the time in seconds, the better the agility and dynamic balance of the participant.

In 2013, Rikli and Jones re-performed reliability and validity tests on items of the Senior Fitness Test. The validity coefficient = 0.87 (n = 75) and test-retest reliability consistency = 0.89 (n = 73) of the 30-second chair stand test and the validity coefficient = 0.79 (n = 73) and the test-retest reliability consistency = 0.90 (n = 71) of the 8-foot up-and-go test indicated that the items in the Senior Fitness Test have high reliability and validity.^[22]^ Physical fitness norms for older adults differ by sex and age. The physical fitness norms in this study were based on those established by the Sports Administration of the Ministry of Education of Taiwan.^[23]^

#### 2.5.5. St. Thomas Risk Assessment Tool in Falling Elderly Inpatients (STRATIFY)

There were 5 items on the STRATIFY: fall (s) in the past year or during hospitalization, unclear consciousness, disorientation, or restlessness (any item), activities of daily living affected by poor vision, need to go to the bathroom frequently (e.g., frequent urination or diarrhea), and no tolerance for activities (only stands for a short time and needs assistance from others or assistive equipment to get out of bed). Each item is scored as follows: 1 point for yes and 0 points for no. The total score ranged from 0 to 5 points. Individuals with STRATIFY scores ≥ 2 points were defined as having a high risk of falls. The sensitivity and specificity of STRATIFY were 92.4% to 93.0% and 68.3% to 87.7%.^[24,25]^

### 2.6. Data analysis

After data collection, data analysis and coding were performed, and the data were documented and statistically analyzed using SPSS 28.0 statistical software (IBM, Armonk). Continuous variables, including age and body mass index (BMI), are presented as means and standard deviations. Categorical variables are presented as frequency distributions and percentages. The main outcomes were MoCA-T score, hand grip strength, 30-second chair stand test time, 8-foot up-and-go test time, and STRATIFY score. The outcomes were analyzed by paired *t* test and effect size to assess the effects of interventions.

### 2.7. Ethical considerations

This study was approved by the Institutional Review Board of Taipei Veterans General Hospital of Taiwan (No. 2021-02-022B) and conducted in accordance with the Declaration of Helsinki. Participants were informed about the objectives of the study, confidentiality policies, and their right to withdraw at any time without incurring a disadvantage. Written informed consent was obtained from all the participants. This study was registered at ClinicalTrials.gov (NCT05558839).

## 3. Results

Forty-one participants completed the 12-week intervention, as well as the pretest and post-test, from April 2021 to January 2022. The attendance rate of the participants was 89%.

### 3.1. Baseline characteristics

Baseline characteristics of the study participants are presented in Table 2. The mean age of the participants was 77.5 years, with the highest proportion ranging from 75 to 84 years (17, 41.5%). There were more females (24, 58.5%) than males. The mean BMI was 22.3 ± 3.02, and 11 participants (26.9%) were overweight (BMI ≥ 24). Half of the participants had mild or moderate dementia, that is, 21 participants had moderate dementia (51.2%) and 20 participants had mild dementia (48.8%). More than half (23, 56.1%) of the participants were married. The most common primary caregivers were sons or daughters (in law) (18, 43.9%), followed by spouses (15, 36.6%). Among the 41 participants, 34 (82.9%) had other chronic diseases in addition to dementia, with hypertension and diabetes mellitus occurring most frequently (12 each, each accounting for 29.3%). Regarding exercise habits, participants who reported that they “did not exercise” (17, 41.5%) accounted for the highest proportion of participants, followed by participants who exercised “1 to 2 times a week” (15, 36.6%).

**Table 2:** Basic attribute information of the older adults with dementia in this study (n = 41).

Characteristics	Mean (SD)/n (%)
Age	77.5 (7.95)
65–74 years	16 (39.0)
75–84 years	17 (41.5)
≥85 years	8 (19.5)
Gender	
Male	17 (41.5)
Female	24 (58.5)
Body mass index (BMI)	22.3 (3.02)
Underweight: BMI < 18.5	4 (9.8)
Normal range: 18.5 ≤ BMI < 24	26 (63.4)
Overweight: 24 ≤ BMI < 27	7 (17.1)
Mild obesity: 27 ≤ BMI < 30	4 (9.8)
Degree of dementia	
Mild dementia (CDR = 1)	20 (48.8)
Moderate dementia (CDR = 2)	21 (51.2)
Educational attainment	
Below elementary school	21 (51.3)
Middle school	6 (14.6)
High school	5 (12.2)
College/university	9 (21.9)
Marital status	
Married	23 (56.1)
Widowed	16 (39.0)
Single	2 (4.9)
Carer relationship	
Spouse	15 (36.6)
Son or daughter (in law)	18 (43.9)
Foreign domestic worker	3 (7.3)
No caregiver	5 (12.2)
Chronic diseases other than dementia	
No	7 (17.1)
Yes	34 (82.9)
Comorbidities	
Hypertension	12 (29.3)
Diabetes mellitus	12 (29.3)
Hyperlipidemia	8 (19.5)
Fall within a month	
No	34 (82.9)
Yes	7 (17.1)
STRATIFY score	
Total score < 2 points	25 (61.0)
Total score ≥ 2 points	16 (39.0)
Exercise habits	
None	17 (41.5)
1–2 times a week	15 (36.6)
3 times or more a week	9 (22.0)

CDR = Clinical Dementia Rating, SD = standard deviation, STRATIFY = St. Thomas risk assessment tool in falling elderly inpatients.

### 3.2. Quantitative outcomes

#### 3.2.1. Cognitive assessment

**Figure 1.**
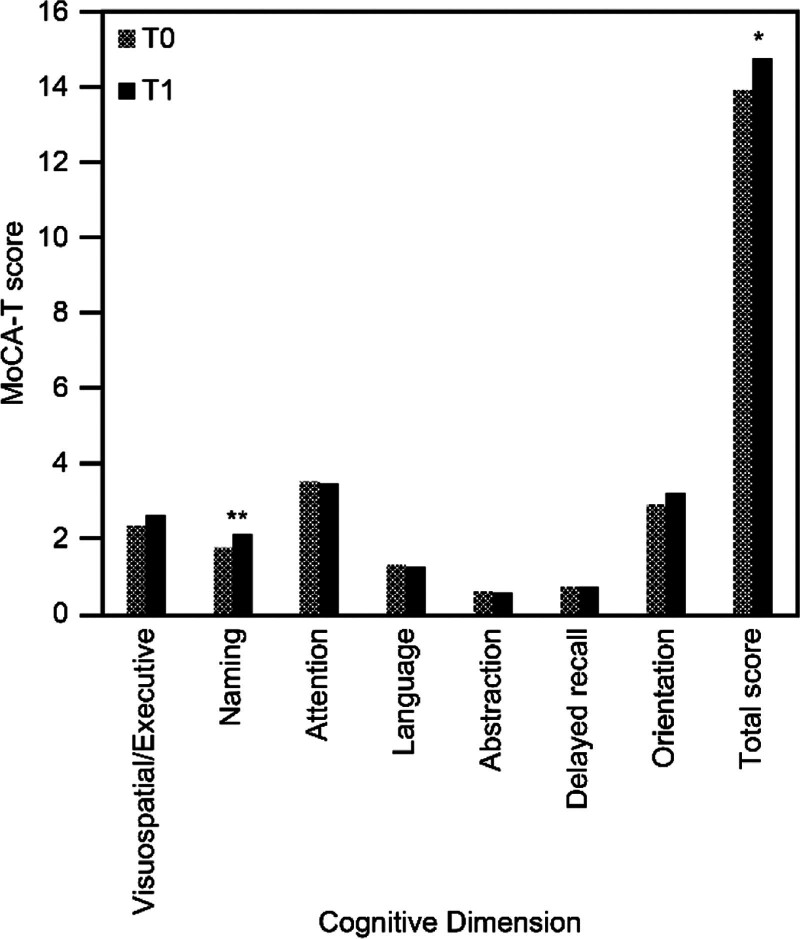
Comparison of cognitive dimensions of the MoCA-T and total MoCA-T scores between pretest and posttest. *P < .05, **P < .01. MoCA-T = Taiwan version of the Montreal Cognitive Assessment, T0 = baseline and pretest, T1 = post-test.

All participants had MoCA-T scores of < 26 points. The pre- and post-test MoCA-T scores for the 41 participants are shown in Table 3 and Figure 1. After the intervention, the mean total MoCA-T score increased significantly by approximately 0.83 points (*P* = .014), with the mean score for “naming (3 points)” increasing significantly by approximately 0.36 points (*P* = .001). The mean scores for “visuospatial execution (5 points),” “delayed memory,” and “orientation” increased slightly, whereas the mean scores for “attention,” “language,” and “abstraction” decreased slightly after the intervention. Participants with both mild (n = 20) and moderate (n = 21) dementia showed an increase in MoCA-T scores, with only those with mild dementia reaching significance (*P* = .031). The overall effect size for cognitive function was 0.402, with values of 0.522 for mild dementia and 0.310 for moderate dementia.

**Table 3:** Pretest and posttest results for the outcome indicators.

Variables	n	Pretest	Posttest	Difference between pretest and posttest (95% CI)				Effect size
		Mean (SD)	Mean (SD)	Lower	Upper	*t*	*P*	
MoCA-T (points)								
Mild dementia	20	17.05 (5.35)	18.00 (5.13)	0.098	1.802	2.334	.031*	0.522
Moderate dementia	21	10.95 (5.64)	11.67 (4.88)	−0.335	1.764	1.420	.171	0.310
Total MoCA-T score	41	13.93 (6.25)	14.76 (5.89)	0.179	1.480	2.577	.014*	0.402
Hand grip strength (kg)								
Male	17	21.3 (8.88)	21.6 (8.63)	−1.128	1.598	0.366	.719	0.089
Female	24	13.4 (4.65)	14.0 (3.92)	−0.699	1.928	0.968	.343	0.198
30-second chair stand test (times)								
Male	17	11.5 (3.46)	11.8 (4.11)	−0.631	1.131	0.605	.554	0.151
Female	24	9.7 (3.67)	10.9 (4.48)	0.152	2.265	2.366	.027*	0.483
8-foot up-and-go test (s)								
Male	17	11.6 (4.11)	11.3 (5.72)	−1.486	0.904	−0.519	.611	0.130
Female	24	12.9 (5.99)	12.1 (5.57)	−1.650	−0.029	−2.142	.043*	0.437
STRATIFY score	41	1.32 (1.15)	1.27 (1.05)	−0.235	0.137	−0.53	.599	0.083

CI = confidence interval, MoCA-T = the Taiwan version of Montreal Cognitive Assessment, SD = standard deviation, STRATIFY = St. Thomas risk assessment tool in falling elderly inpatients.

* *P* < .05.

#### 3.2.2. Physical fitness

**Figure 2.**
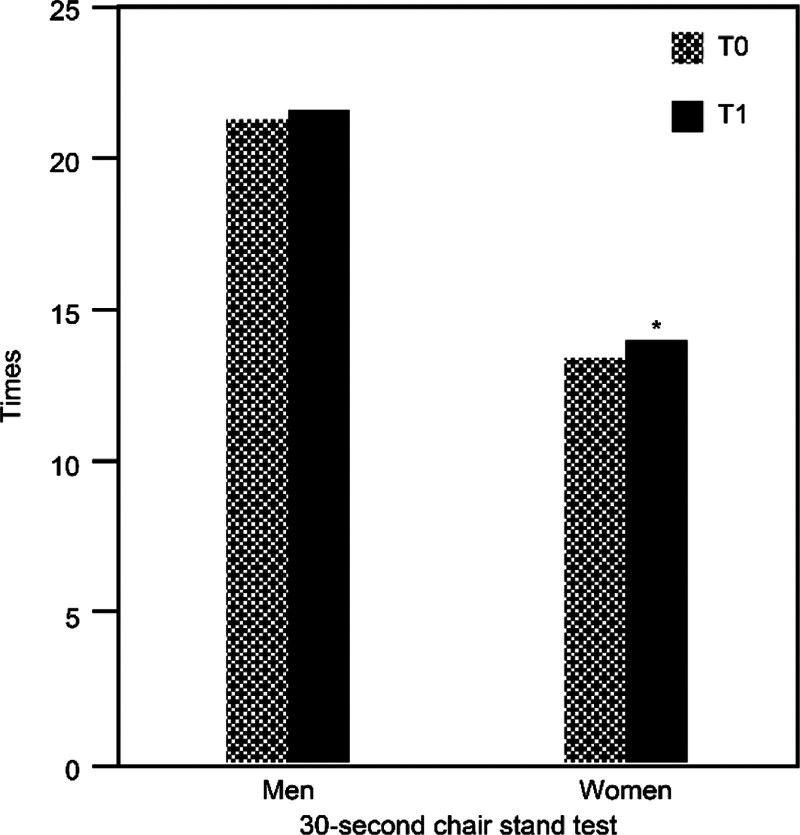
Comparison of the 30-second chair stand test between pretest and posttest. T0 = baseline and pretest, T1 = posttest. *P < .05.

**Figure 3.**
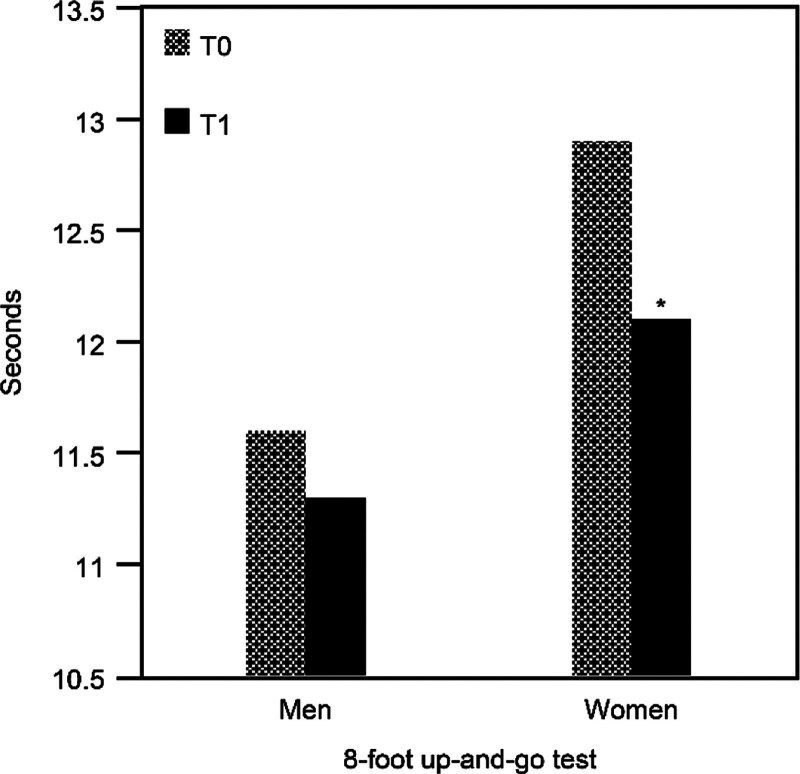
Comparison of the 8-foot up-and-go test between pretest and posttest. T0 = baseline and pretest, T1 = posttest. *P < .05.

For upper limb muscle strength, after the intervention, male participants showed an increase of approximately 0.3 kg in mean grip strength, while female participants exhibited a mean posttest grip strength increase of approximately 0.7 kg. Both male and female participants showed non-significant increases in mean upper limb muscle strength (*P* = .719 for males and *P* = .343 for females) after the intervention (Table 3). For lower limb muscle strength performance, after the intervention, male participants increased their mean 30-second chair stand test score by approximately 0.3 times, while female participants showed a significant increase of approximately 1.2 times (*P* = .027; effect size = 0.483) (Fig. 2). For agility and dynamic balance performance, after the intervention, male participants decreased their mean 8-foot up-and-go test time by approximately 0.2 seconds, while female participants exhibited a significant decrease of approximately 0.8 seconds (*P* = .043; effect size = 0.437) (Fig. 3).

#### 3.2.3. Fall risk assessment

A total of 39.0% of the participants were at a high risk of falls (total STRATIFY score ≥ 2 points), and 7 (17.1%) participants had fallen 1 month before the intervention. The mean STRATIFY score for participants decreased slightly, but not significantly by 0.05 points (*P* = .599), after the intervention (Table 3).

### 3.3. Qualitative outcomes

#### 3.3.1. The cognitive status of the participants improved

In the first few weeks, Ms. Ye, who was 83 years old, frequently asked, “*What time is it? I have something to do at home, and I want to go home*” approximately to 3 to 5 minutes after the start of each class. Other participants helped to reassure her. After several weeks, Ms. Ye’s ability to concentrate in class had improved significantly. Ms. Chen, who was 72 years old, said: “*She* (referring to Ms. Ye) *is doing better after attending class for a while and doesn*’*t keep saying that she needs to go home*.”

#### 3.3.2. The content of interventions was diverse and moderately difficult, stimulating the interest of the participants and increasing their participation

The daughter of 80-year-old Ms. Liu happily shared, “*My mother usually says she wants to take a break, but she told me to take her to class after lunch. I was so touched. She never liked class so much. She usually didn*’*t exercise, but now she always tells me that it is good to play with the gym ball here*.” Mr. Lin, who was 72 years old, had not attended classes at dementia service stations before this study. Mr. Lin’s wife said, “*Now my husband is used to attending class here every Monday. I plan to let him continue to attend classes here after this class ends.*”

#### 3.3.3. Muscle strength training using a gym ball and a resistance band caused participants to sweat and pant slightly, achieving the desired exercise effect

Ms. Zhou, who was 91 years old, said, “*Oh, I usually don*’*t exercise; so, I pant when I do this exercise.*” while drawing figure 8 with both feet using a gym ball. The participant’s movements were correct and rapid.

#### 3.3.4. Investment in and enthusiasm for participation was higher among female participants than male participants

Ms. Wang (90 years), Ms. Liu (80 years), Ms. Zhou (91 years), and Ms. Cai (82 years) attended classes at different study sites. The female participants did not have other chronic diseases and did not usually exercise. They showed great enthusiasm for participating, and their performance in many exercises was better than that of the younger participants, despite their older age. In contrast, the male participants were more passive in learning and rarely expressed personal thoughts.

#### 3.3.5. Participants with mild dementia more easily completed learning tasks than did participants with moderate dementia

Ms. Guo, who was 71 years old and had mild dementia, used a notebook to record each trainer’s name. During the board game class, she often grasped the rules of the game with a brief reminder and guided other participants when playing games. Mr. Lin, who was 72 years old and had moderate dementia, was not accustomed to attending classes at the dementia service stations. When he first started exercise training, he often performed the opposite movements. After several weeks of exercise training, many reminders, and timely praise, he kept up with the movements and moved in the correct direction. Mrs. Lin was willing to let him continue to attend classes because of his improved performance.

### 3.4. Adverse events and safety

One participant, aged 83 years, with a history of hypertension, experienced headaches before the start of exercise. She was immediately assisted in the measurement of vital signs. She could take a break first and then join the class after her discomfort subsided. No other intervention-related adverse events were reported. This study was conducted at community-based dementia service stations where venue safety adhered to government regulations, including procedures for incidents and emergencies. One researcher and primary caregivers accompanied the participants to ensure their safety throughout the study period.

## 4. Discussion

This study aimed to investigate the effects of cognitive-based board games and multi-component exercise interventions on cognitive function, physical fitness, and fall risk in older adults with dementia.

### 4.1. The combined intervention of cognitive-based board games and multi-component exercise has a positive impact on the cognition of older adults with dementia

In this study, 41 participants showed a significant increase in cognitive function scores after the intervention (*P* = .014), with only those with mild dementia reaching significance (*P* = .031). This finding is consistent with the results reported by Anderson-Hanley et al^[16]^ who implemented the interactive physical and cognitive training system (iPACES™) ≥2 times a week for 12 weeks (20 minutes each session), and the results reported by Liu et al^[26]^ who implemented exergaming-based Tai Chi 3 times a week for 12 weeks (50 minutes each session).

While there was a significant increase in cognitive function scores, the average MoCA-T score increased by only 0.83 points. Research indicates that changes within 4 points in MoCA scores fall within the margin of the measurement error.^[27]^ However, effect size is more effective than *P* values in presenting the strength of the relationship between interventions and outcome variables.^[28]^ The overall effect size for cognitive function in this study was 0.402, with values of 0.522 for mild dementia and 0.310 for moderate dementia. According to Cohen’s standards for effect size, values below 0.2 are considered small, those between 0.3 and 0.7 are medium, and those above 0.8 are large.^[14,28]^ The effect size for cognitive function in this study was within the medium range. Furthermore, qualitative data indicated that both participants and primary caregivers perceived improvements in some participants’ cognitive functions. These findings support the effectiveness of the intervention based on both the effect size and qualitative data. However, there was only a minimal increase in MoCA-T scores. Future research should increase the sample size and incorporate control groups to obtain more comprehensive evidence to evaluate effectiveness.

### 4.2. The interventions significantly improved lower limb strength, agility, and dynamic balance among older adults with dementia

This study demonstrated that cognitive-based board games and multi-component exercise interventions significantly improved lower limb strength, agility, and dynamic balance among older adults with dementia. This finding is consistent with the results reported by Lemke et al^[13]^ who implemented exercise and cognitive dual-task training twice a week for 10 weeks (1.5 hours each time), the results reported by Karssemeijer, Bossers et al^[17]^ who implemented exercise and cognitive somatosensory game training 3 times a week for 12 weeks (30–40 minutes each time), and the results reported by Park et al^[29]^ who implemented a dual-task exercise program once a week for 24 weeks (110 minutes each session).

The effect size for females in the 30-second chair stand test was 0.483, and for the 8-foot up-and-go test, it was 0.437. The effect size for female lower limb performance fell within the medium range. During the study, female participants seemed to be more engaged in physical activities and had a higher degree of cooperation and enthusiasm than male participants. In addition, the participants interacted and communicated with each other and were able to remind themselves and learn from each other, providing a potential explanation for the greater (significant) improvement in lower limb muscle strength, agility, and dynamic balance performance in female participants than in male participants. This finding is consistent with the results reported by Chen et al^[30]^ who implemented a 12-week exercise class (once a week for 60 minutes) for 30 older adults with mild and moderate dementia.

### 4.3. The fall risk of older adults with dementia needs long term evaluation

In this study, the fall risk scores decreased slightly but not significantly after 12 weeks of intervention, similar to the findings by Nyman et al^[31]^ who implemented a 45-minute Tai Chi exercise once a week for 20 weeks for 85 older adults with mild to moderate dementia in a UK community. In this study, the average age of the 41 participants was 77.5 years old, and among them, 34 (82.9%) had comorbidities, all of which were known risk factors for falls in older adults with dementia. These findings highlight the high risk of falls among study participants and underscore the importance of muscle strength training for fall prevention. In this study, we hypothesized that the mechanism by which cognitive-based board games and multi-component exercise interventions reduce falls would involve an improvement in physical fitness performance. Although we did not observe a significant reduction in fall risk scores, the tracking of fall risk assessments after the intervention may not have been sufficiently long. Therefore, follow-up periods of 6 months, 1 year, or longer are needed to assess fall risk.

### 4.4. Feasibility of evidence-based board game and exercise intervention modules

This study explored the feasibility and benefits of cognitive-based board games and multi-component exercise interventions in older adults with dementia. At present, exercise or cognitive interventions at community-based dementia service stations in Taiwan are mostly in the form of group recreation, and the content of interventions lacks rigorous planning and design. This study integrated cognitive interventions into different types of board games to allow older adults to have interpersonal interactions through groups, resulting in more fun, thus increasing their willingness to attend class. Gym balls and resistance bands are convenient and safe training tools for older adults with dementia. During this study, the participants sweated and panted slightly, achieving the desired exercise effect, and their physical performance improved. Therefore, the participants and caregivers were more willing to participate in the class. The physical activity and fitness performance of older adults with dementia are usually much worse than those of older adults with mild cognitive impairment. Appropriate resistance training can increase muscle mass, muscle strength, and bone density and improve balance and physical function.^[30]^ The cognitive and exercise interventions in this study are applicable to older adults with different degrees of dementia, as evidenced by the implementation of interventions at community-based dementia service stations.

Intervention fidelity refers to faithful and correct implementation of components within a defined intervention. It is monitored through measurements based on the manual that describes adherence to the intervention components. Manuals are used to train and supervise interventionists for research and practical purposes. Supervision involves observing intervention sessions in person through recordings, or in response to interventionist process recordings.^[32]^ In this study, manuals were developed to guide trainers in accurately executing components, dosages, and protocols. Furthermore, one researcher supervised the trainers to ensure adherence to the manual and recorded the operational process, thus ensuring consistency and fidelity of the interventions. Intervention fidelity is commonly described with 5 dimensions amenable to measurement: adherence, dosage, quality of intervention delivery, participant responsiveness, and program differentiation.^[33]^ The present study focused solely on adherence, dosage, and quality of intervention delivery. Future research should measure fidelity using all 5 dimensions to ensure the effectiveness of the intervention.

Therefore, interventions that combine cognitive-based board games with multi-component exercises can be widely promoted among older adults living in both community and institutional settings, especially those with mild cognitive impairment or dementia. Such interventions aim to delay the decline in cognitive or physical function, and further reduce the risk of falls.

## 5. Limitations

This study was conducted at 5 community-based dementia service stations in northern Taiwan, and only 41 participants were included through convenience sampling. It is important to note that due to the limited number of participants and the specific characteristics of the study population, which presented challenges in recruitment, the findings of this study may not be generalizable to older adults with dementia from different geographic regions.

The absence of a control group in this study, attributed to the special nature of the study participants and the difficulties encountered in participant recruitment, may limit the ability to draw definitive conclusions regarding the effectiveness of the interventions. Therefore, for future research endeavors in this area, a control group should be incorporated into the study design to provide a basis for comparison and enhance the validity of the results.

In Taiwan, the norms for physical fitness assessments vary between males and females. As a result, when participants were categorized by sex, the sample size for each subgroup decreased. This reduction in sample size may have affected the statistical power for hypothesis testing, highlighting the need for a larger sample size to ensure reliable and robust results.

## 6. Conclusions

This study aimed to investigate the feasibility and effect of cognitive-based board games and multi-component exercise interventions on cognitive function, physical fitness, and fall risk in older adults with dementia. The results indicated that the cognitive function and physical fitness of the participants improved, and that lower limb strength, agility, and dynamic balance performance significantly improved in female participants. Cognitive-based board games and multi-component exercise interventions have been shown to maintain and improve cognitive function and physical fitness among older adults with dementia, thereby delaying dementia-related disabilities. Persistent muscle strength training can prevent and reduce the risk of fall. Group activities can also promote interpersonal relationships and interactions in older adults with dementia. Institutions and communities should promote interventions for older adults with mild cognitive impairment or dementia.

## Author contributions

**Conceptualization:** Hui-Wen Chang, Guey-Hau Wu.

**Data curation:** Hui-Wen Chang.

**Formal analysis:** Hui-Wen Chang.

**Investigation:** Hui-Wen Chang, Guey-Hau Wu.

**Methodology:** Guey-Hau Wu.

**Project administration:** Guey-Hau Wu.

**Resources:** Guey-Hau Wu.

**Supervision:** Guey-Hau Wu.

**Validation:** Guey-Hau Wu.

**Visualization:** Guey-Hau Wu.

**Writing – original draft:** Hui-Wen Chang.

**Writing – review & editing:** Hui-Wen Chang, Guey-Hau Wu.

## References

[1] Taiwan Alzheimer’s Disease Association. Getting to know Alzheimer’s disease [in Chinese]. 2023. http://www.tada2002.org.tw/About/IsntDementia. Accessed July 20, 2023.

[2] World Health Organisation. Global Status Report on the Public Health Response to Dementia. WHO; 2021.

[3] Çinar N Şahiner TAH. Effects of the online computerized cognitive training program BEYNEX on the cognitive tests of individuals with subjective cognitive impairment and Alzheimer’s disease on rivastigmine therapy. Turk J Med Sci. 2020;50:231–8. 31887854 10.3906/sag-1905-244 PMC7080370

[4] Yang Y Kwak YT. Improvement of cognitive function after computer-based cognitive training in early stage of Alzheimer’s dementia. Dement Neurocogn Disord. 2017;16:7–11. 30906364 10.12779/dnd.2017.16.1.7 PMC6427986

[5] Iuliano E di Cagno A Cristofano A. Physical exercise for prevention of dementia (EPD) study: background, design and methods. BMC Public Health. 2019;19:659. 31142290 10.1186/s12889-019-7027-3 PMC6542067

[6] Gauthier S Webster C Servaes S Morais JA Rosa-Neto P. World Alzheimer report 2022: life after diagnosis: navigating treatment, care and support. Alzheimer’s Disease International; 2022.

[7] Brett L Stapley P Meedya S Traynor V. Effect of physical exercise on physical performance and fall incidents of individuals living with dementia in nursing homes: a randomized controlled trial. Physiother Theory Pract. 2019;37:38–51. 30912690 10.1080/09593985.2019.1594470

[8] Chen KH Chen HH Li L Lin H Chen CL Chen NC. The impact of exercise on patients with dementia: a 2-year follow-up. Medicine (Baltimore). 2020;99:e20597. 32502032 10.1097/MD.0000000000020597 PMC7306297

[9] Rees J Burton A Walters K Cooper C. Exploring the provision and support of care for long-term conditions in dementia: a qualitative study combining interviews and document analysis. Dementia (London, England). 2023;22:820–37. 36883009 10.1177/14713012231161854 PMC9996169

[10] Ministry of Health and Welfare. Dementia care and service plan [in Chinese]. Updated January, 2023. https://1966.gov.tw/LTC/cp-6456-70024-207.html. Accessed May 8, 2023.

[11] Ching-Teng Y. Effect of board game activities on cognitive function improvement among older adults in adult day care centers. Soc Work Health Care. 2019;58:825–38. 31432758 10.1080/00981389.2019.1656143

[12] Karssemeijer EGA Aaronson JA Bossers WJR Donders R Rikkert MGMO Kessels RPC. The quest for synergy between physical exercise and cognitive stimulation via exergaming in people with dementia: a randomized controlled trial. Alzheimers Res Ther. 2019;11:3. 30611286 10.1186/s13195-018-0454-z PMC6320611

[13] Lemke NC Werner C Wiloth S Oster P Bauer JM Hauer K. Transferability and sustainability of motor-cognitive dual-task training in patients with dementia: a randomized controlled trial. Gerontology. 2019;65:68–83. 30041173 10.1159/000490852

[14] Kotrlik JW Williams HA. The incorporation of effect size in information technology, learning, and performance research. Inf Technol Learn Perform J. 2003;21:1–7.

[15] American College of Sports Medicine. ACSM’s Guidelines for Exercise Testing and Prescription. 10th ed. Lippincott Williams & Wilkins; 2017.

[16] Anderson-Hanley C Stark J Wall KM. The Interactive Physical and Cognitive Exercise System (iPACES™): effects of a 3-month in-home pilot clinical trial for mild cognitive impairment and caregivers. Clin Interv Aging. 2018;13:1565–77. 30233154 10.2147/CIA.S160756 PMC6130272

[17] Karssemeijer EGA Bossers WJR Aaronson JA Sanders LMJ Kessels RPC Rikkert MGMO. Exergaming as a physical exercise strategy reduces frailty in people with dementia: a randomized controlled trial. J Am Med Dir Assoc. 2019;20:1502–8. 31409559 10.1016/j.jamda.2019.06.026

[18] Hsieh CY Wang HH. Effect of multicomponent exercises program intervention on functional fitness and cognitive function in elderly with mild cognitive impairment [in Chinese]. NCYU Phys Educ Health Recreat J. 2017;16:13–32.

[19] Nasreddine ZS Phillips NA Bédirian V. The Montreal Cognitive Assessment, MoCA: a brief screening tool for mild cognitive impairment. J Am Geriatr Soc. 2005;53:695–9. 15817019 10.1111/j.1532-5415.2005.53221.x

[20] Tsai CF Lee WJ Wang SJ Shia BC Nasreddine Z Fuh JL. Psychometrics of the Montreal Cognitive Assessment (MoCA) and its subscales: validation of the Taiwanese version of the MoCA and an item response theory analysis. Int Psychogeriatr. 2012;24:651–8. 22152127 10.1017/S1041610211002298

[21] Wei YC Chen CK Lin C. Normative data of mini-mental state examination, montreal cognitive assessment, and Alzheimer’s disease assessment scale-cognitive subscale of community-dwelling older adults in Taiwan. Dement Geriatr Cogn Disord. 2022;51:365–76. 35820405 10.1159/000525615 PMC9677874

[22] Rikli RE Jones CJ. Development and validation of criterion-referenced clinically relevant fitness standards for maintaining physical independence in later years. Gerontologist. 2013;53:255–67. 22613940 10.1093/geront/gns071

[23] Sports Administration of the Ministry of Education. Complete fitness guide for people above 65 years [in Chinese]. 2020. https://isports.sa.gov.tw/apps/Download.aspx?SYS=TIS&MENU_CD=M04&ITEM_CD=T02&MENU_PRG_CD=2&ITEM_PRG_CD=7. Accessed January 17, 2021.

[24] Oliver D Britton M Seed P Martin FC Hopper AH. Development and evaluation of evidence-based risk assessment tool (STRATIFY) to predict which elderly inpatients will fall: Case-control and cohort studies. BMJ. 1997;315:1049–53. 9366729 10.1136/bmj.315.7115.1049 PMC2127684

[25] Silva SO Barbosa JB Lemos T Oliveira LAS Ferreira ADS. Agreement and predictive performance of fall risk assessment methods and factors associated with falls in hospitalized older adults: a longitudinal study. Geriatr Nurs. 2023;49:109–14. 36495792 10.1016/j.gerinurse.2022.11.016

[26] Liu CL Cheng FY Wei MJ Liao YY. Effects of exergaming-based Tai Chi on cognitive function and dual-task gait performance in older adults with mild cognitive impairment: a randomized control trial. Front Aging Neurosci. 2022;14:761053. 35370622 10.3389/fnagi.2022.761053 PMC8965318

[27] Feeney J Savva GM O’Regan C King-Kallimanis B Cronin H Kenny RA. Measurement error, reliability, and minimum detectable change in the mini-mental state examination, montreal cognitive assessment, and color trails test among community living middle-aged and older adults. J Alzheimers Dis. 2016;53:1107–14. 27258421 10.3233/JAD-160248

[28] Tomczak M Tomczak E. The need to report effect size estimates revisited. An overview of some recommended measures of effect size. Trends Sport Sci. 2014;1:19–25.

[29] Park H Park JH Na HR. Combined intervention of physical activity, aerobic exercise, and cognitive exercise intervention to prevent cognitive decline for patients with mild cognitive impairment: a randomized controlled clinical study. J Clin Med. 2019;8:940. 31261796 10.3390/jcm8070940 PMC6678908

[30] Chen HH Lin PH Yang SY Lee YC. Effect of physical activity on physical fitness for day-care elderly with dementia [in Chinese]. J Taiwan Occupat Therap Res Pract. 2018;14:101–10.

[31] Nyman SR Ingram W Sanders J. Randomised controlled trial of the effect of Tai Chi on postural balance of people with dementia. Clin Interv Aging. 2019;14:2017–29. 31819385 10.2147/CIA.S228931 PMC6875562

[32] Santacroce SJ Maccarelli LM Grey M. Intervention fidelity. Nurs Res. 2004;53:63–6. 14726779 10.1097/00006199-200401000-00010

[33] An M Dusing SC Harbourne RT Sheridan SM; START-Play Consortium. What really works in intervention? Using fidelity measures to support optimal outcomes. Phys Ther. 2020;100:757–65. 31944249 10.1093/ptj/pzaa006

